# Effect of the Presence of Cardiac Tamponade on Jugular Vein Diameter in Cases of Cardiac Arrest Due to Thoracic Aortic Disease

**DOI:** 10.7759/cureus.50791

**Published:** 2023-12-19

**Authors:** Michika Hamada, Hiroki Nagasawa, Hiroaki Taniguchi, Tatsuro Sakai, Hiromichi Ohsaka, Kazuhiko Omori, Youichi Yanagawa

**Affiliations:** 1 Acute Critical Care Medicine, Juntendo University Shizuoka Hospital, Izunokuni, JPN

**Keywords:** cardiac arrest, computed tomography, aortic dissection, jugular vein, cardiac tamponade

## Abstract

Background

There have so far been no reports regarding whether or not the jugular veins remain distended even in cases of cardiac arrest, which is the worst form of shock. We focused on the diameter of the jugular vein in neck computed tomography (CT) in cases of thoracic aortic disease resulting in cardiac arrest to determine whether or not cardiac tamponade increased the diameter.

Methodology

From January 2014 to December 2021, patients were eligible for inclusion when they were transported to our hospital, judged to be in cardiac arrest at the emergency department, and then diagnosed with thoracic aortic disease as the cause of cardiac arrest according to CT. Patients were divided into two groups according to the presence (tamponade (+)) or absence (tamponade (-)) of cardiac tamponade. Comparisons between the two groups were also conducted after excluding cases in which relief of cardiac tamponade was obtained before CT or that had hemothorax.

Results

There were 52 cases in the cardiac tamponade (+) group and 16 in the cardiac tamponade (-) group. The diameters of both the right and left internal jugular veins were significantly larger in the cardiac tamponade (+) group than in the cardiac tamponade (-) group. After excluding cases with relief of cardiac tamponade before CT and hemothorax complications, the right and left internal and external jugular vein diameters in the cardiac tamponade (+) group were still significantly greater than those in the cardiac tamponade (-) group.

Conclusions

The present study showed that the cardiac tamponade induced by thoracic aortic cases tended to display larger internal jugular vein diameters compared to cases without cardiac tamponade, even in patients experiencing cardiac arrest. Additionally, cardiac tamponade consistently presented with larger diameters in the right-sided jugular vein.

## Introduction

In cases of cardiac arrest, we routinely investigate the cause by ultrasonography, and unless the patient is known to have terminal cancer or the family does not wish for it, blood tests and computed tomography (CT) are performed [1-5]. The causes of cardiac arrest include thoracic aortic diseases, some of which can lead to cardiac arrest via cardiac tamponade. Cardiac tamponade might cause obstructive shock leading to cardiac arrest, which then causes distension of the external jugular vein due to impaired venous return [6-9].

However, the diameter of the external jugular vein is linked to that of the internal jugular vein, which is sometimes used as an indicator of circulating blood volume [10,11]. There have so far been no reports regarding whether or not the jugular veins remain distended even in cases of cardiac arrest, which is the worst form of shock. In addition, there have been no reports measuring the jugular vein diameter during cardiac arrest itself. Therefore, in this study, we focused on the diameter of the internal jugular vein during CT in cases of thoracic aortic disease resulting in cardiac arrest.

## Materials and methods

The study was approved by the Ethics Committee of Juntendo University Shizuoka Hospital (approval number: 401).

Our hospital is located in eastern Shizuoka Prefecture and is the base hospital for the eastern Shizuoka Prefecture Doctor Helicopter. Although there are two emergency centers in eastern Shizuoka, one does not have an exclusive working emergency physician, so most critically ill patients are transported to this hospital, including those with cardiac arrest [12,13]. The target population is approximately 1.2 million people.

Patients were eligible for inclusion when they were transported to our hospital by ambulance or helicopter, judged as having endogenous cardiac arrest in the emergency department, and then diagnosed with thoracic aortic disease as the cause of cardiac arrest according to CT between January 2014 and December 2021. The definition of endogenous excluded trauma, suffocation, overdose, accidental hypothermia, and drowning. The exclusion criteria included cases without cardiac arrest, those involving exogenous cardiac arrest, and endogenous cardiac arrest with the stipulation that the cause was not thoracic aortic disease.

**Figure 1:**
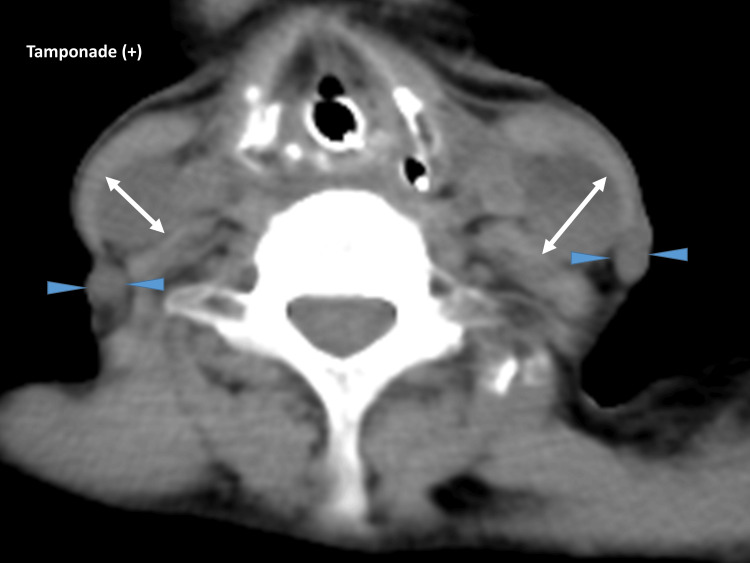
Neck computed tomography. Example of the measurement of the diameter of the right and left internal jugular vein at the vocal cord portion in a patient with cardiac tamponade (arrowhead, external jugular vein; arrow, internal jugular vein).

The following data were collected: sex, age, thoracic aortic disease classification (Stanford A, Stanford B, or ruptured thoracic aortic aneurysm), fibrinogen/fibrin degradation products (FDP) level, maximum diameter of the right and left internal and external jugular vein at the vocal cord portion utilizing software integrated with an electronic medical chart system (Figure 1), outcome (survived or dead), the presence or absence of witnesses [14], bystander cardiopulmonary resuscitation (CPR), initial rhythm, return of spontaneous circulation (ROSC), cardiac tamponade, chest opening before CT, and hemothorax. Initially, patients were divided into two groups according to the presence (tamponade (+)) or absence (tamponade (-)) of cardiac tamponade, and a comparative study was performed. The definition of cardiac tamponade included cardiac arrest accompanied by confirmed pericardial effusion through ultrasound or CT, a criterion debated among multiple physicians during the conference as a potential cause for cardiac arrest. Secondary, as part of the subgroup examination, comparisons between the two groups were also conducted after excluding cases in which relief of cardiac tamponade was obtained before CT or that had hemothorax, as cardiac tamponade relief and hemothorax were thought to decrease the jugular vein diameter. To predict the presence of tamponade, an appropriate cutoff value for the diameter of the internal jugular vein was analyzed. The receiver operating characteristic curve was employed to identify the cutoff value, defined as the point where the Youden index (sensitivity + specificity - 1) reaches its maximum. Further, the external jugular vein diameter measurements were also compared at the same measurement level as the internal jugular vein diameter. Finally, a comparative analysis was conducted to examine the differences between the left and right internal jugular vein and external jugular vein in cases of cardiac tamponade.

Statistical methods included a non-paired Student’s t-test, chi-square, and contingency table test, with statistical significance set at p < 0.05. Data are presented as mean ± standard deviation.

## Results

From January 2014 to December 2021, 14,559 patients were transported to our department and treated. Of these, 1,772 were cases of cardiac arrest, with an endogenous origin in 1,335 cases. Sixty-eight cases of endogenous thoracic aortic disease were diagnosed through CT imaging and thus included in this study (52 in the cardiac tamponade (+) group and 16 in the cardiac tamponade (-) group). Initially, 68 patients experienced cardiac arrest upon arrival at the emergency room. Eight patients achieved ROSC and subsequently underwent a CT examination. The remaining 60 patients underwent CT examination. Among them, three patients achieved ROSC after the CT examination.

The results of analyses between the two groups are shown in Table 1. There were no marked differences between the two groups regarding gender, age, bystander CPR, initial rhythm, emergency thoracotomy, ROSC, FDP values, or outcomes. There was a significant difference in the percentage of classification subgroups of thoracic aortic disease between the two groups. The proportion of witnesses and hemothorax was significantly lower in the tamponade (+) group than in the tamponade (-) group. In contrast, the diameters of both the right and left internal jugular veins were significantly larger in the tamponade (+) group than in the tamponade (-) group.

**Table 1:** Analysis findings. n.s.: not significant; PEA: pulseless electrical activity

Variables	Unit or variable description	Tamponade (+)	Tamponade (-)	P-value
		(n = 52)	(n = 16)	
Sex	Male	19	5	n.s.
	Female	33	11	
Age	Years	75.3 ± 15.4	78.0 ± 14.4	n.s.
Classification	Aneurysmal rupture	0	3	0.001
	Stanford A	52	12	
	Stanford B	0	1	
Witnessed collapse	Yes	20	11	0.03
	No	32	5	
Bystander CPR	Yes	16	6	n.s.
	No	36	10	
Initial rhythm	Asystole	19	7	n.s.
	PEA	33	9	
Hemothorax	Yes	13	2	<0.0001
	No	39	13	
Emergency thoracotomy	Yes	16	2	n.s.
	No	36	14	
Return of spontaneous circulation	Yes	8	3	n.s.
	No	44	16	
Diameter of right jugular vein	mm	17.6 ± 5.3	12.1 ± 6.7	<0.001
Diameter of left jugular vein	mm	12.8 ± 4.7	8.1 ± 3.8	<0.001
Fibrin degradation product	μg/mL	324.2 ± 339.6	422.9 ± 381.1	n.s.
Outcome	Survival	1	1	n.s.
	Fatal	51	15	

**Figure 2:**
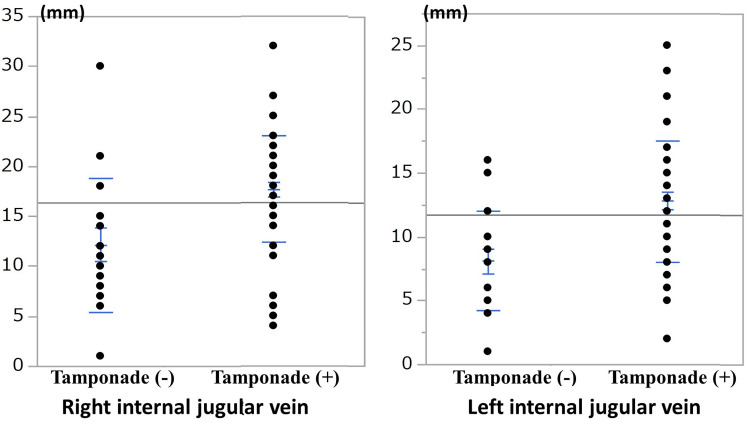
Distribution of the right and left internal jugular vein diameters. The diameters of both the right and left internal jugular veins were significantly larger in the tamponade (+) group than in the tamponade (-) group.

The distribution of the right and left internal jugular vein diameters is shown in Figure 2. The cutoff values for the tamponade (+) group were right 15.0 mm (sensitivity 80.7 %, specificity 75.0%) and left 10.0 mm (sensitivity 78.0 %, specificity 75.0 %).

Table 2 shows the results of the comparison between the two groups after excluding cases of cardiac tamponade relief and hemothorax complications. There were no marked differences between the two groups regarding gender, age, disease category, witnesses, bystander CPR, initial waveform, ROSC, FDP values, or outcomes. However, both the right and left internal jugular vein diameters were significantly dilated in the tamponade (+) group compared with the tamponade (-) group.

**Table 2:** Results of the analysis of cases without hemothorax and emergency thoracotomy. n.s.: not significant; PEA: pulseless electrical activity; JV: jugular vein; CPR: cardiopulmonary resuscitation

Variables	Unit or variable description	Tamponade (+)	Tamponade (-)	P-value
		(n = 26)	(n = 3)	
Sex	Male/Female	10	1	n.s.
	Female	16	2	
Age	Years	75.7 ± 14.3	76.0 ± 14.4	n.s.
Classification	Stanford A	26	3	n.s.
Witnessed collapse	Yes	9	2	n.s.
	No	17	1	
Bystander CPR	Yes	7	1	n.s.
	No	19	2	
Initial rhythm	Asystole	11	1	n.s.
	PEA	15	2	
Return of spontaneous circulation	Yes	2	1	n.s.
	No	24	2	
Diameter of right internal JV	mm	20.1 ± 3.6	10.3 ± 1.5	<0.01
Diameter of left internal JV	mm	13.5 ± 4.3	8.6 ± 0.5	0.02
Diameter of right external JV	mm	6.7 ± 3.0	1.0 ± 1.0	0.01
Diameter of left external JV	mm	4.9 ± 2.1	1.0 ± 1.0	0.02
Fibrin degradation product	μg/mL	228.9 ± 295.8	479.3 ± 478.0	n.s.
Outcome	Survival	1	1	n.s.
	Fatal	25	2	

**Figure 3:**
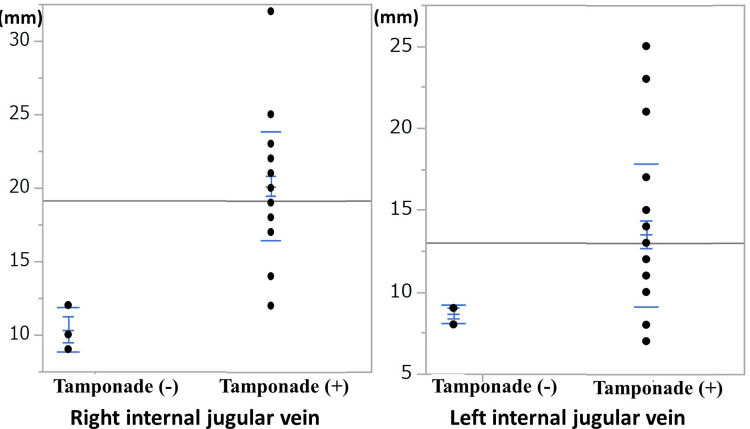
Distribution of the right and left internal jugular vein diameters after excluding cases of cardiac tamponade relief and hemothorax complications. The diameters of both the right and left internal jugular veins were significantly larger in the tamponade (+) group than in the tamponade (-) group.

The distribution of the right and left internal jugular vein diameters is shown in Figure 3. The cutoff values for the tamponade (+) group were right 14.0 mm (sensitivity 96.1%, specificity 100%) and left 10.0 mm (sensitivity 88.4%, specificity 100%).

**Figure 4:**
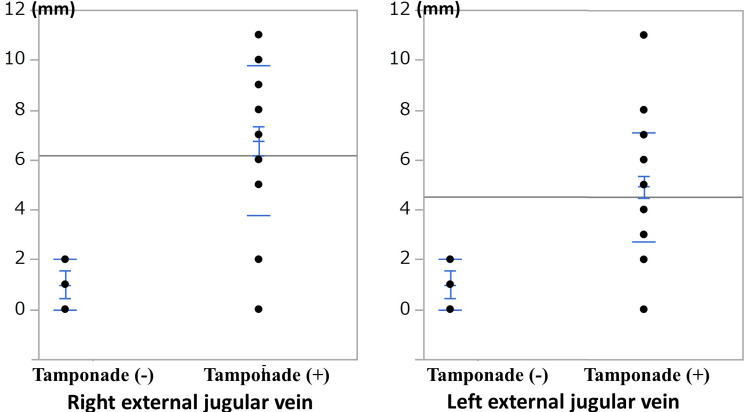
Distribution of the right and left external jugular vein diameters after excluding cases of cardiac tamponade relief and hemothorax complications. The diameters of both the right and left external jugular veins were also significantly larger in the tamponade (+) group than in the tamponade (-) group.

The diameters of the both right and left external jugular veins were also significantly larger in the tamponade (+) group than in the tamponade (-) group (Figure 4).

In cases of cardiac tamponade, the average jugular vein diameters were statistically significantly larger on the right compared to the left side for all cases of the internal jugular vein (17.6 versus 12.8 mm, p < 0.0001), cases excluding those with open chest or hemothorax for the internal jugular vein (20.1 versus 13.5 mm, p < 0.0001), and the external jugular vein (6.7 versus 4.9, p = 0.01).

## Discussion

This is the first report to suggest that the presence of cardiac tamponade contributes to the widening of the internal jugular vein diameter, even in cases of cardiac arrest due to thoracic aortic disease. Widening of the jugular vein in the setting of tamponade is a well-known phenomenon. However, to our knowledge, there have been no reports of persistent jugular vein widening even in patients who have experienced cardiac arrest. Additionally, the diameter of the right jugular vein was larger than that of the left in cases of cardiac tamponade.

Previous reports have suggested that the internal jugular vein diameter is useful for estimating the circulating blood volume, central venous pressure, and blood pressure response to transfusion and for evaluating heart failure and the prognosis of pulmonary artery thromboembolism [15-18]. Cardiac tamponade has also been found to increase central venous pressure from restrictive disturbance [19]. For this reason, we speculated that even in cases of cardiac arrest, the elevated central venous pressure might have remained and contributed to the widening of the internal jugular vein diameter. If the enlargement of the internal jugular vein diameter is confirmed by ultrasound in patients with cardiac arrest, physicians should carefully evaluate whether cardiac tamponade exists or not to facilitate spontaneous circulation through decompression.

Why would physicians need a CT scan-derived cutoff for an indirect finding (jugular vein distension) if the CT scan already provides the final diagnosis of thoracic aortic disease with hemopericardium? The study’s findings suggest potential applications of ultrasound examinations, especially in identifying cardiac tamponade resulting from thoracic aortic diseases. The consistent dilation of internal jugular veins, particularly on the right side, could function as an ultrasound marker, offering a non-invasive and valuable diagnostic tool for clinicians assessing patients suspected of tamponade due to thoracic aortic diseases before resorting to CT scans. We attempted key methods to promptly identify patients experiencing cardiac arrest due to cardiac tamponade, aiming for the immediate relief of the tamponade. This was necessary as ultrasound frequently proved ineffective in detecting tamponade caused by coagulation, if there is a lack of careful observation or if physicians are inexperienced [1,3]. If the presence of cardiac tamponade can be clarified, even in cases of cardiac arrest, decompression can be performed in expectation of ROSC, and the number of patients able to return to society can be improved [2]. Therefore, resuscitation methods including relief of cardiac tamponade may be required if physicians detect dilation of the internal or external jugular vein in patients with endogenous cardiac arrest.

The design of the present study exhibits several limitations. First, it was a retrospective study, the number of cases studied was small, the cases were cardiac arrest cases, and cases with high venous pressure due to other diseases (e.g. pulmonary embolism or cor pulmonale) were included. The results may lack generalizability to a broader population, given the specificity of the inclusion criteria. The findings are primarily relevant to individuals with thoracic aortic disease who experience cardiac arrest and meet the defined criteria. In addition, the results imply that recognizing jugular vein distension during resuscitation might not justify introducing additional procedures. Second, the decision to exclude cases without cardiac arrest, those with exogenous cardiac arrest, and cases of endogenous cardiac arrest with a cause other than thoracic aortic disease introduces subjectivity and interpretation challenges. Third, there may have been anatomical variations in the internal jugular vein [20]. Lastly, subgroup analyses, such as excluding cases with relief of cardiac tamponade before CT or cases with hemothorax, may result in smaller sample sizes and reduced statistical power. Whether or not the present findings can be applied to all cases of cardiac arrest, including those with endogenous disease, requires further investigation of a large number of prospective cases.

## Conclusions

The present study showed that cardiac tamponade induced by thoracic aortic cases tended to display larger internal jugular vein diameters compared to cases without cardiac tamponade, even in patients experiencing cardiac arrest. Additionally, cardiac tamponade consistently presented with larger diameters in the right-sided jugular vein.
